# TCD4^pos^ lymphocytosis in rheumatoid and psoriatic arthritis patients following TNFα blocking agents

**DOI:** 10.1186/s12967-017-1135-6

**Published:** 2017-02-21

**Authors:** Andrea Picchianti Diamanti, Bruno Laganà, Maria Christina Cox, Emanuela Pilozzi, Rachele Amodeo, Maurizio Bove, Milica Markovic, Roberta Di Rosa, Simonetta Salemi, Maria Laura Sorgi, Maria Manuela Rosado, Raffaele D’Amelio

**Affiliations:** 1 Department of Clinical and Molecular Medicine, S. Andrea University Hospital, School of Medicine and Psychology, “Sapienza” University, Via di Grottarossa 1039, 00189 Rome, Italy; 2 Haematology Unit, Rome, Italy; 3 UOC Anatomic Pathology, Rome, Italy; 4 UOC Laboratory Diagnostic, Rome, Italy; 5 Menarini Group, Research Toxicology Center (R.T.C), Pomezia, Italy

**Keywords:** Rheumatoid arthritis, Psoriatic arthritis, Lymphocytosis, TCR, Anti-TNF-α agent

## Abstract

**Background:**

Lymphocyte expansion and true lymphocytosis are commonly observed in the everyday clinical practice. The meaning of such phenomenon is often poorly understood so that discrimination between benign and malignant lymphocytosis remains difficult to establish. This is mainly true when lymphocytosis rises in patients affected by immune-mediated chronic inflammatory diseases under immunosuppressive treatment, conditions potentially associated with lymphomagenesis. In this brief report the development of mild T CD4^pos^ lymphocytosis in a group of patients with chronic arthritis under anti-TNF-α treatment is described.

**Methods:**

Two hundred eight rheumatoid arthritis (RA) and psoriatic arthritis (PsA) patients have been evaluated longitudinally for at least 1-year before and 2-years after anti-TNF-α therapy introduction for the possible appearance of a lymphocyte expansion. In patients who developed lymphocyte expansion, T, B and NK cells were analysed.

**Results:**

Twenty-five out of 208 (12%) subjects developed a mild T CD4^pos^ lymphocytosis, during anti-TNF-α therapy, which reverted after its interruption. Higher lymphocyte count, more frequent use of steroids and shorter disease duration, before biological therapy start, have emerged as risk factors for lymphocytosis development.

**Conclusions:**

This is the first longitudinal cohort study evaluating the onset of lymphocytosis in RA and PsA patients under anti-TNF-α treatment and its possible clinical relevance. A mild T CD4^pos^ lymphocytosis has been observed in 12% of RA and PsA patients probably related to anti-TNF-α treatment as previously reported by anecdotal cases. Patients with higher baseline lymphocyte count, use of steroids and shorter disease duration before the introduction of biologic therapy, seem to be prone to develop this laboratory reversible abnormality.

## Background

Lymphocyte expansion and true lymphocytosis are commonly features in the everyday clinical practice. The meaning of such phenomenon is frequently poorly clear, so that discrimination between benign and malignant lymphocytosis is often hard to establish. Thus, the search for markers helping in such discrimination is highly warranted. This is mainly felt when lymphocyte abnormalities arise during the course of an immune-mediated chronic inflammatory disease, such as rheumatoid arthritis (RA) and psoriatic arthritis (PsA) under immunosuppressive treatment, all conditions which predispose to lymphomagenesis. Lymphocyte expansion may roughly be divided into either reactive, reversible and benign phenomenon or a malignant lymphoproliferative, irreversible, disorder. Despite the causes of these abnormalities not always come to light, infections are frequently involved as promoting factor. Infectious agents generally induce reactive lymphocyte expansion, but some of them, including Epstein Barr Virus (EBV) or Kaposi’s Sarcoma Herpesvirus (KSHV), Human T lymphotropic virus 1 (HTLV1), Human immune deficiency virus (HIV), Hepatitis C virus (HCV) [1], *Helicobacter pylori* [2], *Borrelia burgdorferi* [3], *Chlamydophila psittaci* [4], *Campylobacter jejuni* [5], *Achromobacter xylosoxidans* [6], may be the direct cause of malignant lymphoproliferative disorders. The diagnostic discrimination between the above reported conditions (mainly the recognition of the malignant lymphoproliferation) [7] is not always easy, because of the lack of reliable lymphomagenesis predictive markers.

An increased risk for lymphoproliferative disorders has been reported in RA and PsA patients, with most of the studies indicating higher risk for Hodgkin’s and non-Hodgkin’s lymphomas (HL and NHL) [8–10]. Several factors can be implicated in the onset or maintenance of lymphomagenesis in RA and PsA, such as the genetic background and the persistent stimulation of T and B cells by unknown antigens. Continuous immune stimulation leads to chronic inflammation and imbalance between inflammatory and regulatory cytokines [11, 12]. Also immunosuppressive therapies might be associated to an increased risk of lymphoma [13]. Increased malignancy rate has been initially reported, in both diseases, in association to anti-TNF-α therapy, but this observation is still controversial because data has not been confirmed by recent meta-analyses [14–18]. To date, four case reports of either T CD4^pos^ or CD8^pos^ lymphocytosis with an immunophenotype of large granular lymphocytes (LGL) [19, 20] and one case of T CD4^pos^ lymphocyte polyclonal expansion [21] in RA patients under anti-TNF-α therapy have been described.

In the daily clinical activity we too have observed lymphocyte expansion in RA and PsA patients under immunosuppressive therapy. These patients have been therefore systematically analysed, in order to possibly infer the clinical meaning of such observation.

## Methods

Two hundred eight subjects (140 RA and 68 PsA), poorly responding to methotrexate (Mtx) thus even receiving an anti-TNF-α agent, have been followed-up for the level of peripheral blood lymphocytes. Lymphocytosis was defined as a number of circulating lymphocytes ≥3500/µl for at least 3 months [22–24]. Patients have been selected among those attending S. Andrea University Hospital Immuno-Rheumatology outpatient clinic from December 2010 to November 2015. Patients have been evaluated longitudinally for at least 1-year before and 2-years after addition of biologic therapy, the influence of which could therefore be analysed in every patient, who served as control of himself. In patients with circulating lymphocytes ≥3500/µl, peripheral blood (PB) was collected and lymphocyte subpopulations (total T cells [CD3^pos^], T helper [CD3^pos^CD4^pos^CD45^pos^], T cytotoxic [CD3^pos^CD8^pos^CD45^pos^], natural killer [CD16^pos^CD56^pos^CD45^pos^] and B cells [CD19^pos^CD45^pos^]) were analysed using a standard protocol based on four-color immune-fluorescence flow-cytometer, as previously described [25]. In brief, BD multitest IMK kit with tricount tubes (BD biosciences) consisting of a four-color direct immunofluorescence reagent kit for FACS Canto II (BD biosciences) to determine mature human lymphocyte subsets in erythrocyte-lysed whole blood samples was used. The following monoclonal antibodies were present in different combinations: FITC-labeled CD3, PE-labeled CD8, PerCP-labeled CD45, APC-labeled CD4, PE-labeled CD16 and FITC-labeled CD56, APC-labeled CD19. Twenty thousand lymphocytes were tested for each sample. Data was analysed using a dedicated Canto II software.

Data was analysed using StatView statistical program for MacIntosh (StatView Software, San Diego, CA) and P value was determined with the paired Student’s t test. P values <0.05 were considered to be statistically significant.

## Results

**Fig. 1:**
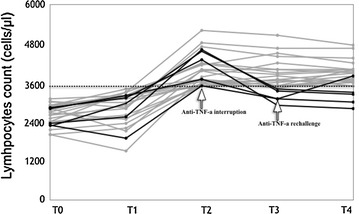
Total lymphocyte count (cells/µl) in 25 patients with lymphocytosis throughout the follow-up. *Dashed line* indicates the upper limit of normal lymphocyte count. *T0* Methotrexate ± steroids therapy; *T1* Addition of anti-TNF-a therapy; *T2* Peak of lymphocytes level (*bold black lines* identify the patients in whom anti-TNF-a therapy was interrupted); *T3* Three months from T2; *T4* Twelve months from T2

None of the 208 total patients developed lymphocytosis during the 1-year treatment with Mtx (7.5–15 mg/weekly) ± low dose steroids (<7.5 mg of prednisone/daily). After start of anti-TNF-α treatment, in combination with preexisting synthetic immunosuppressants, 25/208 (12%, 15 RA and 10 PsA, Group A) showed a significant increase in the lymphocyte count leading to a mild lymphocytosis [from 2800 to 4000 mean cells/µl (mean ratio of increase 1.44); *P* < *0.001*; Fig. 1], whereas in the remaining 183 patients (Group B) lymphocyte count remained substantially stable (from 1866 to 2012 [mean ratio of increase 1.1]) (Table 1). Of note, no changes in immunosuppressive therapy other than biologic introduction were adopted in the group A patients at least in the 3 months before the lymphocytosis onset.

**Table 1:** Main demographic and clinical parameters of the patients who developed (25; group A) and did not develop (183; group B) TCD4^pos^ lymphocytosis at baseline

N. pts	Age (years)	M/F	RA/PsA	DAS28	Disease duration (years)	Biological N (%)					ccs use (%)	Lymphocyte count (cells/μl)
						Etn	Ada	Ifx	Gol	Cert		
25	58 ± 13	9/16	15/10	6.2	4.2 ± 5.6*	11 (44)	7 (28)	4 (16)	3 (12)	0	60^^^	2800 ± 499^✝^
183	59.6 ± 14	59/124	116/67	5.6	8.2 ± 6.9	98 (54)	53 (29)	15 (8)	12 (6)	5 (3)	40	1866 ± 362

Values are expressed as mean ± SD

*Etn* etanercept, *Ada* adalimumab, *Ifx* infliximab, *Gol* golimumab, *Cert* certolizumab, *CCS* corticosteroids

* P < 0.05 (group A vs group B)

^^^P < 0.05 (group A vs group B)

^✝^P < 0.001 (group A vs group B)

The median time between the beginning of the biological therapy and the development of lymphocytosis was 9 months (range 2–24 months; 16/25 patients within 6 months). No concomitant clinical signs of infection were present nor serological tests for hepatitis B, C, cytomegalovirus and EBV or screening tests for tuberculosis were positive.

One hundred nine patients received etanercept (Etn), 60 adalimumab (Ada), 19 infliximab (Ifx), 15 golimumab (Gol) and 5 certolizumab pegol (Ctz). Eleven out of 109 (10%) under Etn, 7/60 (11.7%) under Ada, 4/19 (21%) under Ifx and 3/15 (20%) patients under Gol developed lymphocyte expansion (no significant differences).

A comparison of the main clinical and demographic characteristics of group A and B before starting anti-TNF-α treatment was performed, aiming at identifying possible distinctive parameters to be used as predictive markers for lymphocytosis. The analysis showed that group A patients had a significantly shorter disease duration before the beginning of the biological therapy and more frequent use of steroids (Table 1). Furthermore, group A patients presented physiological, but significantly higher, lymphocyte count as compared to group B (2800 vs 1866 cells/µl; *P* < *0.001*). The disease activity score (DAS) instead did not show any significant difference between the two groups. The analysis of PB lymphocyte subsets revealed that B and NK cells were not the cause of the lymphocyte expansion. In fact, the distribution of these two lymphocyte subpopulations was at the lowest limit of the normal range (9.6% ± 1.4 and 8% ± 2.3; normal 10–16 and 8–14% respectively). In contrast, an increase in the mean percentage and number of the total CD3^pos^ cells (79.5 ± 5.6%; 3120 ± 220/μl) and CD3^pos^CD4^pos^ cells (50.8 ± 4.4%; 1962 ± 190/μl) accompanied by a mild reduction in the CD3^pos^CD8^pos^ T cells (27.2 ± 1.2%; 1028 ± 60/μl) was observed, with a consequent CD4/CD8 ratio at the upper limit of the normal range. In order to gain more insights on this T cell expansion, a TCRγ gene rearrangement analysis was performed in all 25 patients. In five individuals the predominance of a monoclonal peak of about 160 bp was observed. Following hematologist suggestion a total lymph nodes ultrasonography was performed. Although the analysis did not reveal any sign of lymph node or spleen enlargement, treatment with biologic therapy was interrupted as precautionary measure. Within 3 months after anti-TNF-α therapy suspension, total lymphocyte count returned to normal values (from 4134 ± 506 to 3168 ± 203/µl; *P* < *0.008*, Fig. 1) and remained stable over 9 further months of follow-up. In one of these patients, in which a rechallenge with biologic therapy was necessary as a consequence of RA flare up, it was possible to observe the reappearance of lymphocytosis. In the group A patients, who continued biologic therapy, lymphocyte count remained stably above normal values during the 1-year period of follow-up (Fig. 1).

## Discussion

A group of RA and PsA patients who developed a mild TCD4^pos^ lymphocytosis during anti-TNF-α treatment is here described. Higher lymphocyte count, use of steroids in association to Mtx and shorter disease duration, before the beginning of the biologic therapy, were associated with the onset of TCD4^pos^ lymphocytosis. Considering that DAS was similar in the two groups of patients, the significant higher lymphocyte count observed in the group A may be more reliable expression than DAS of active/aggressive disease, as documented by the more frequent use of steroids and significant shorter disease duration before starting biologic therapy.

In the absence of clear guidelines for the management of lymphocytosis in RA and PsA patients under anti-TNF-α therapy, the biologic therapy was interrupted in the five patients presenting a lymphocytosis and a monoclonal TCRγ chain repertoire. Within 3 months following the interruption of TNF-α inhibitor a significant decrease in the lymphocytes level, until physiological values, was observed, whereas in the patients who did not interrupt biologic therapy the number of lymphocytes remained stable (Fig. 1). In one of these patients, in which a rechallenge with biologic therapy was necessary as a consequence of RA flare up, it was possible to observe the reappearance of lymphocytosis. Analysis of the data with Naranjo algorithm gave a probability score result for causality of 6 thus indicating a probable anti-TNF-α induced event [26]. Despite the persistence of lymphocyte expansion and the probable link with anti-TNF-α agents, as previously reported in anecdotal cases (19–21), none of the patients developed clinical or laboratory signs of progression toward malignancy at 1-year follow-up. Consequently, this haematologic abnormality appears as a benign reversible drug-related laboratory finding other than a serious adverse event, at least for the observation period. In RA patients, the risk of developing hematologic tumors is higher than in the healthy population [27]. In particular large granular lymphocytosis is frequently associated with RA and other autoimmune diseases [28]. Reversible T lymphoproliferation in RA patients under anti-TNF-α therapy has been occasionally described in case reports. Sometimes the number of CD8^pos^ T cells is altered [20], but more frequently CD4^pos^ is the main T cell subset to be affected. The lymphocyte expansion may be either monoclonal [19] or polyclonal [21]. Reversible lymphoproliferation has also been described as a consequence of CMV reactivation [29]. However, the cause for reversible lymphoproliferation in these patients has not yet been identified, it is known that TNF-α can limit T cell expansion and induce apoptosis in both naïve and memory T cells [30], consequently TNF-α blocking agents may lead to a dysregulation of these biologic mechanisms, thus resulting in T cell expansion and/or prolonged survival. Moreover, in addition to anti-TNF-α biologic treatment, the role of steroids in promoting lymphoproliferation can not be forgotten. Recent studies have demonstrated that steroids are able to significantly reduce circulating T regulatory cells [31], thus adding fuel to the hypothesis that immunosuppressive therapy may in certain conditions induce T cell expansion. The higher risk of lymphoma in RA patients treated with TNF-α inhibitors has been suggested but not clearly demonstrated. Several factors may hinder a definitive correlation between anti-TNF-α therapy and lymphoma development, among which the selection of the population enrolled in different studies or national registries and the inter individual variability of the immune system exposed to TNF-α. In fact, being a double sword tool, TNF-α may play a defensive role by stimulating NK and cytotoxic T lymphocytes, but can also be “offensive” acting as mediator of cancer development through chronic inflammation promotion [13]. Thus, in theory, TNF-α antagonists may either promote or inhibit cancer growth [32]. The onset of B-cell lineage lymphomas and hepatosplenic T cell lymphoma (HSTCL) has been observed in patients with Crohn’s disease who received anti-TNF-α agents mainly in association to thiopurines [33–35]. Of note, in patients with psoriasis, an absolute lymphocytosis associated to γδ T cell lymphoma after treatment with anti-TNF-α has been observed [36, 37].

## Conclusions

This is the first longitudinal cohort study evaluating the onset of lymphocytosis in RA and PsA patients under anti-TNF-α treatment and its possible clinical relevance. A mild T CD4^pos^ lymphocytosis has been observed in a not negligible percentage of RA and PsA patients probably related to anti-TNF-α treatment as previously reported by anecdotal cases. Higher lymphocyte count, use of steroids and shorter disease duration before the introduction of biologic therapy, have come to light as risk factors for lymphocytosis development. Despite the persistence of lymphocyte expansion and its probable link with anti-TNF-α agents, none of the patients developed clinical or laboratory signs of progression toward malignancy at 1-year follow-up. Although current data are reassuring and suggest that this alteration have a benign trend, a longer follow-up and genetically analysis of these patients is suitable in order to gain a deeper insight of this phenomenon.

## Abbreviations

RA: rheumatoid arthritis
PsA: psoriatic arthritis
TNF: tumor necrosis factor
Mtx: methotrexate
HL: Hodgkin’s lymphoma
NHL: non-Hodgkin’s lymphoma
PB: peripheral blood
Etn: etanercept
Ada: adalimumab
Ifx: infliximab
Gol: golimumab
Ctz: certolizumab pegol
LGL: large granular lymphocytes
HSTC: hepatosplenic T cell lymphoma
